# Protective Effect of Marine Peptide from *Netunea arthritica cumingii* Against Gentamicin-Induced Hair Cell Damage in Zebrafish

**DOI:** 10.3390/md22110519

**Published:** 2024-11-16

**Authors:** Hongbao Zheng, Ranran Zhu, Yun Zhang, Kechun Liu, Qing Xia, Peihai Li, Xiaoyue Sun, Chen Sun, Shanshan Zhang

**Affiliations:** 1 Biology Institute, Qilu University of Technology (Shandong Academy of Sciences), Jinan 250103, China; 15305412704@163.com (H.Z.); zr00701@163.com (R.Z.); xiaohan_0818@163.com (Y.Z.); hliukch@sdas.org (K.L.); xiaq@sdas.org (Q.X.); lipeihaihg@sina.com (P.L.); 13854398191@163.com (X.S.); 2 Key Laboratory for Drug Screening Technology of the Shandong Academy of Sciences, Jinan 250103, China

**Keywords:** marine peptide, hair cell, zebrafish, *Netunea arthritica cumingii*, otoprotective effects

## Abstract

Auditory hair cell damage induced by aminoglycoside antibiotics (AmAn) leads to hearing loss, which has a serious effect on people’s mental and physical health. This ototoxicity is thought to be related with the excessive accumulation of reactive oxygen species (ROS) in hair cells. However, therapeutic agents that protect hair cells are limited. Marine peptides have been shown to have excellent potential applications in disease prevention and treatment. Therefore, this study investigated the protective effects of an active peptide from *Neptunea arthritica cumingii* against AmAn-induced hair cell damage using the model of hair cell damage zebrafish. We identified the number, ultrastructure, and function of hair cells using fluorescence probes and scanning electron microscopy. The uptake of AmAn, ROS level, mitochondrial permeability transition pore, and apoptosis in hair cells were also tested by fluorescence labeling and TUNEL assay. The molecular mechanism for hair cell protection exerted by the peptide was detected by a real-time quantitative PCR assay. The results indicated that the peptide suppressed the uptake of AmAn but did not damage the function of hair cells mediating hearing. It also prevented ROS accumulation, decreased the occurrence of apoptosis, and rescued the abnormal opening and expressions of mitochondrial permeability transition pore and genes related to antioxidants. The peptide may be an effective therapeutic agent for AmAn-induced ototoxicity. In the future, we plan to use mammalian models to further investigate the otoprotective effect of the peptide.

## 1. Introduction

Nearly 2.5 billion people worldwide will be living with some degree of hearing loss by 2050. At least 700 million of these people will require access to hearing care unless action is taken [1]. Our ability to hear is precious. Hearing loss has a serious effect on people’s ability to study or communicate; it also affects people’s mental health. The loss of auditory hair cells is a common feature of hearing impairment [2]. Auditory hair cells are the mechanosensory cells of the mammalian inner ear that can convert sound stimuli into electrical signals to mediate hearing. Unfortunately, these mammalian cells are terminally differentiated, and once they are lost, they cannot be regenerated spontaneously [3].

Aminoglycoside antibiotics (AmAn) are a potent group of broad-spectrum antibiotics that are frequently used to treat critical Gram-negative infections. However, it is well known that AmAn such as gentamicin (Gen), neomycin and kanamycin can damage auditory hair cells. Currently, the exact mechanism of AmAn-induced hair cell loss remains elusive, but many studies have indicated that it may be related to the excessive reactive oxygen species (ROS) in hair cells induced by AmAn [4,5]. The mitochondria have been identified as a major source of ROS [6]. In other words, compounds that exhibit strong ROS-scavenging ability or protective effect for the mitochondria may be potentially used in the field of preventive therapy for hair cell loss caused by AmAn.

Peptides derived from marine organisms are crucial bioactive natural products. Recent evidence has suggested that marine peptides often exhibit antioxidant, anti-inflammatory, neuroprotective, and antimicrobial activities [7,8]. Therefore, they have high potential medicinal and nutraceutical values. A previous study in our laboratory indicated that an active peptide, YSQLENEFDR (Tyr-Ser-Gln-Leu-Glu-Asn-Glu-Phe-Asp-Arg), from the meat of marine snail *Neptunea arthritica cumingii* (*Nac*) exhibited good antioxidant activity [9]. Hence, we hypothesized that this peptide may also protect auditory hair cells from AmAn-induced damage.

Zebrafish (*Danio rerio*) is an ideal model system for studying auditory hair cells [10]. Similar to mammals, it has inner ear auditory hair cells. In addition, zebrafish also has hair cells on its body surface, called lateral line neuromast hair cells. The morphology and function of lateral line neuromast hair cells in zebrafish are similar to those of mammalian auditory hair cells. They are also highly susceptible to damage caused by AmAn. Moreover, lateral line neuromast hair cells in zebrafish are relatively mature by 5 days post-fertilization (dpf) and are easily visualized using fluorescent dyes [10,11,12].

In summary, to verify our hypothesis, hair cell damage in zebrafish was induced using Gen, which is a type of aminoglycoside antibiotic, for the first time. Then, peptide YSQLENEFDR was evaluated for its potential to alleviate hair cell damage in Gen-treated zebrafish. In addition, the underlying mechanisms were investigated.

## 2. Results

### 2.1. Peptide YSQLENEFDR Treatment Alleviated Gen-Induced Hair Cell Damage

**Figure 1:**
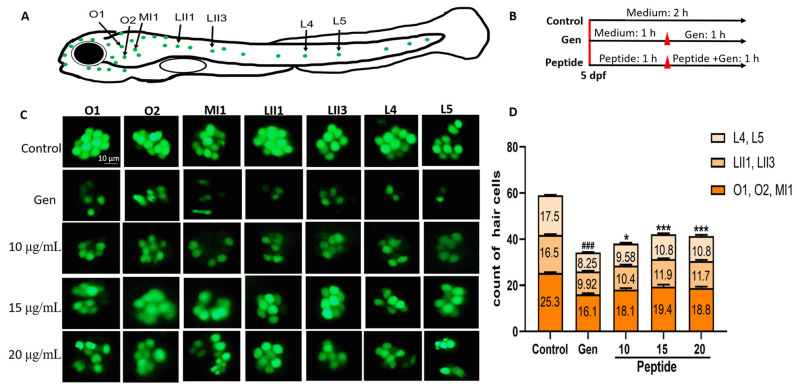
Effect of the peptide on Gen-induced hair cell loss. (**A**) Schematic illustration of lateral line neuromast hair cells. (**B**) Overview of the combined application of peptide YSQLENEFDR and Gen on zebrafish. (**C**) The hair cells were stained with YO-Pro-1 (scale bar = 10 μm). (**D**) Statistical analysis of the hair cell counts in each group (*n* = 12 per group). The number in the bar graph indicates the average number of hair cells in lateral line neuromast. Compared with the control group, ### *p* < 0.001; compared with the Gen-only-treated group, * *p* < 0.05 and *** *p* < 0.001. Gen, gentamicin.

**Figure 2:**
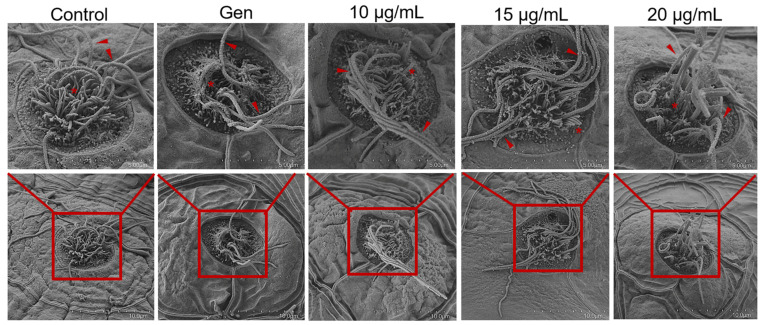
Restorative effect of the peptide on defects in the ultrastructure of hair cells. The red arrow indicates the kinocilia of hair cells; the red asterisk indicates the stereocilia of hair cells.

To assess the otoprotective effects of peptide YSQLENEFDR, 5-dpf zebrafish larvae were exposed to YO-Pro-1, which can highlight living hair cells [13]. As presented in Figure 1, Gen exposure for 1 h significantly reduced the number of hair cells in seven neuromasts (O1, O2, MI1, LII1, LII3, L4, and L5) compared with that in the untreated group. Interestingly, compared with the group treated with only Gen, the group treated with both Gen and peptide YSQLENEFDR (10, 15, and 20 μg/mL) exhibited a significant hair cell count increase (Figure 1C,D). In addition, changes in the ultrastructure of hair cells were observed. As presented in Figure 2, the group that was treated with only Gen exhibited damage in the kinocilia and stereocilia, such as loss or fusion. However, peptide treatment recovered the injured hair cells and reversed them to a state similar to their original conformation. These results indicated that peptide YSQLENEFDR may play a protective role against AmAn-induced hair cell damage.

### 2.2. Pre-Treatment with Peptide YSQLENEFDR Reduced the Uptake of Gen in Hair Cells

**Figure 3:**
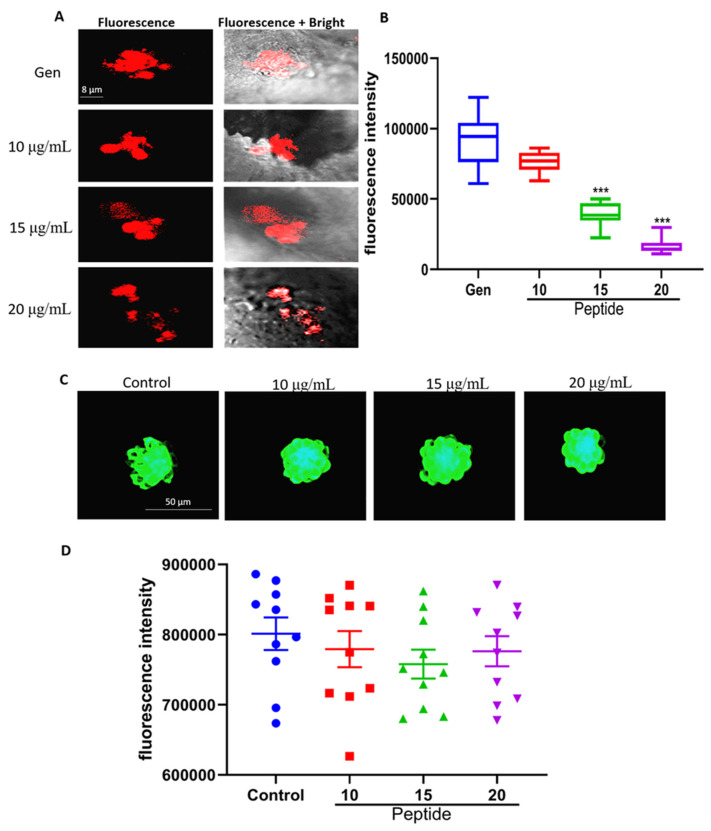
Effects of the peptide on entry of Gen and function of the MET channel in hair cells. (**A**) Representative fluorescence images of GTTR in hair cells. (**B**) Statistical analysis of the fluorescence intensities of GTTR in hair cells (*n* = 10 per group; scale bar = 8 μm). Compared with the Gen-only-treated group, *** *p* < 0.001. (**C**) Representative fluorescence images of FM1-43 in hair cells. (**D**) Statistical analysis of the fluorescence intensities of FM1-43 in hair cells (*n* = 10 per group; scale bar = 50 μm). Gen, gentamicin; MET, mechanoelectrical transducer; GTTR, Gen–Texas Red.

The cellular entry of AmAn is a key factor in hair cell damage. Therefore, we tested the uptake of Gen in hair cells using Gen–Texas Red (GTTR) conjugates. GTTR is the Gen tagged with fluorophore Texas Red. Its fluorescence intensity indicates the content of Gen [14]. As presented in Figure 3A,B, there were many GTTR conjugates in the hair cells of Gen-only-treated group. In contrast, peptide YSQLENEFDR treatment under certain concentrations significantly decreased the fluorescence intensity. These results suggested that the peptide from *Nac* may be an effective therapeutic agent for AmAn-induced ototoxicity by suppressing the uptake of antibiotics.

Many lines of evidence have suggested that mechanoelectrical transducer (MET) channels located on the tips of hair cells are the chief routes by which AmAn enter hair cells. In addition, these channels are also essential for transducing acoustic stimuli into electrical signals [15,16]. Based on these, we assessed the effects exerted by peptide YSQLENEFDR on the activity of MET channels using FM1-43. FM1-43 is a vital fluorescent dye which can specifically enter hair cells through MET channels. This means that the amount of FM1-43 in hair cells can indicate the activity of MET channels [17]. To our surprise, the activity of MET channels was not significantly altered by peptide-only treatment compared with that in the untreated group (Figure 3C,D). These results implied that peptide from *Nac* did not damage the function of hair cells mediating hearing.

### 2.3. Peptide YSQLENEFDR Restrained ROS Accumulation Induced by Gen in Hair Cells

**Figure 4:**
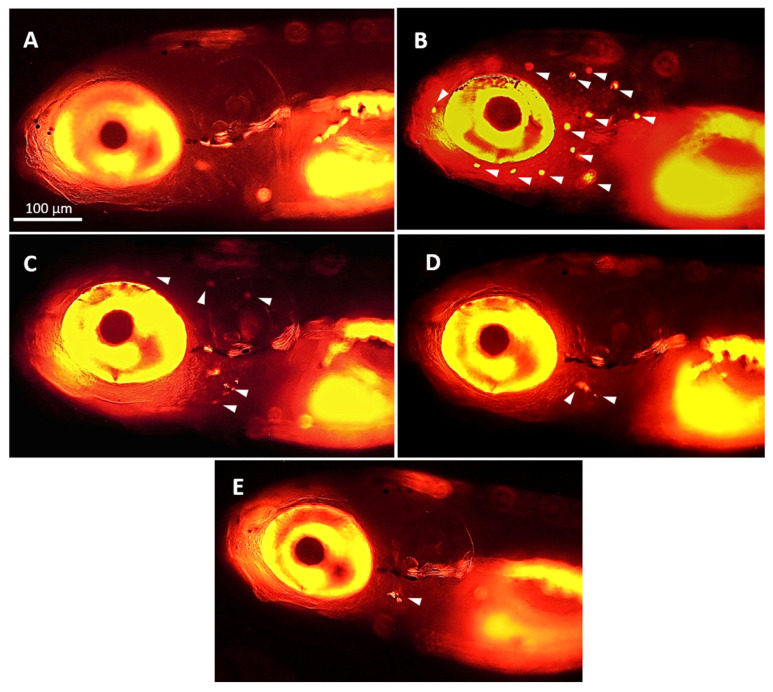
Inhibition of the peptide on ROS accumulation in hair cells. ROS in hair cells were labeled by fluorescence (white arrows). (**A**) Zebrafish larva was not treated with Gen or the peptide. (**B**) Zebrafish larva was treated with only Gen. (**C**) Zebrafish larva was treated with both Gen and 10 μg/mL peptide. (**D**) Zebrafish larva was treated with both Gen and 15 μg/mL peptide. (**E**) Zebrafish larva was treated with both Gen and 20 μg/mL peptide (scale bar = 100 μm). ROS, reactive oxygen species; Gen, gentamicin.

Next, we assessed the ROS level in hair cells. As presented in Figure 4, the Gen-only-treated group exhibited a ROS level higher than that in the untreated group. However, the group treated with both Gen and different concentrations of peptide YSQLENEFDR exhibited reduced ROS levels compared with those in the Gen-only-treated group. Our results indicated that peptide YSQLENEFDR had a strong scavenging ability for ROS in hair cells induced by AmAn.

### 2.4. Peptide YSQLENEFDR Suppressed the Gen-Induced Apoptosis in Hair Cells

**Figure 5:**
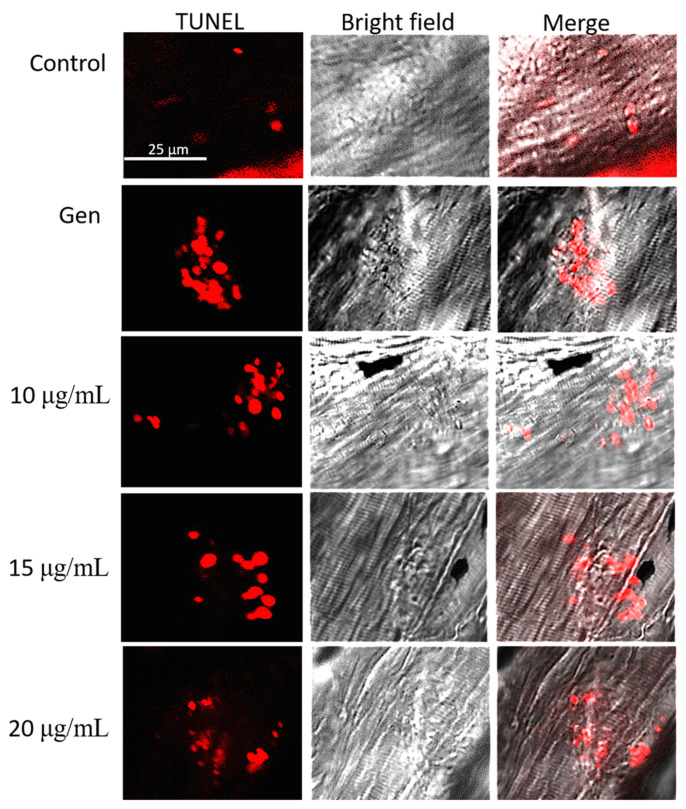
Inhibition of the peptide on apoptosis in hair cells. Apoptotic hair cells were stained with red fluorescence (scale bar = 25 μm).

We found that many apoptotic hair cells appeared in the zebrafish larvae of the Gen-only-treated group. In contrast, no evident apoptotic hair cells were observed in the untreated group. Different concentrations of peptide YSQLENEFDR significantly reduced the number of apoptotic hair cells in the zebrafish larvae (Figure 5). These results suggested that peptide YSQLENEFDR suppressed hair cell apoptosis induced by AmAn in zebrafish.

### 2.5. Peptide YSQLENEFDR Inhibited the Gen-Induced Mitochondrial Permeability Transition Pore Opening

**Figure 6:**
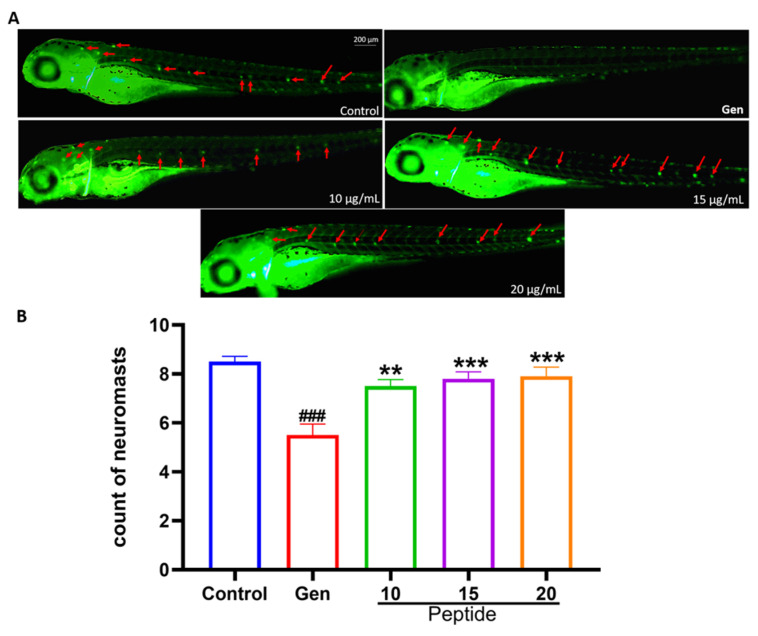
Effect of the peptide on mPTP in hair cells. (**A**) The state of mPTP in the hair cells is indicated by calcein (red arrows). When mPTP was closed, calcein was loaded onto the mitochondria, resulting in the presentation of green fluorescence. When mPTP was open, calcein flowed out of the mitochondria into the cytoplasm, resulting in the disappearance of green fluorescence (scale bar = 200 μm). (**B**) Quantitative assay of the neuromasts exhibiting green fluorescence (*n* = 10). Compared with the control group, ### *p* < 0.001; compared with the Gen-only-treated group, ** *p* < 0.01 and *** *p* < 0.001. mPTP, mitochondrial permeability transition pore.

The mitochondrial permeability transition pore (mPTP) is a key regulator of mitochondrial homeostasis. Sustained mPTP opening will lead to mitochondrial dysfunction and cause apoptosis [18]. Hence, to further explore the protective mechanism of peptide YSQLENEFDR against AmAn-induced hair cell damage, we identified the mPTP opening in zebrafish. Our results indicated that the administration of Gen significantly induced mPTP opening in hair cells. In contrast, treatment with the peptide (10, 15, and 20 μg/mL) remarkably repaired the abnormal mPTP opening (Figure 6). These results implied that peptide YSQLENEFDR protected the mitochondria in hair cells.

### 2.6. Peptide YSQLENEFDR Rescued the Abnormal Expressions of Genes Related to Antioxidation

**Figure 7:**
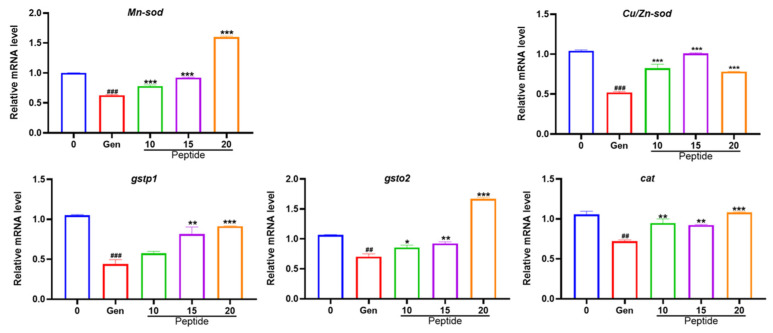
Transcriptional alterations of genes. The amount of gene expression is exhibited as the relative expression. Compared with the control group, ## *p* < 0.01 and ### *p* < 0.001; compared with the Gen-only-treated group, * *p* < 0.05, ** *p* < 0.01, and *** *p* < 0.001.

Oxidative stress is implicated in a pathogenic role in AmAn-induced hair cell damage [19]. Therefore, we assayed the expressions of antioxidation-related genes to investigate the possible underlying mechanism that peptide YSQLENEFDR protects hair cells. The results revealed that transcript levels of *Mn-sod*, *Cu/Zn-sod*, *gstp1*, *gsto2*, and *cat* were significantly downregulated in the zebrafish of the Gen-only-treated group compared with the control, whereas when the peptide reached a certain con-centration, it reversed the downregulation (Figure 7).

## 3. Discussion

Hearing impairment due to AmAn-induced hair cell damage is a common disability and generally causes serious social problems. Therapeutic drugs protecting hair cells are urgently required. Marine peptides are natural products obtained from marine organisms, exhibiting various bioactivities and have excellent potential applications in disease prevention and treatment. In this context, this study focused on the protective effect and mechanism of marine peptides against AmAn-induced hair cell damage. We found that Gen-induced hair cell damage was effectively alleviated by peptide YSQLENEFDR from *Nac*.

**Figure 8:**
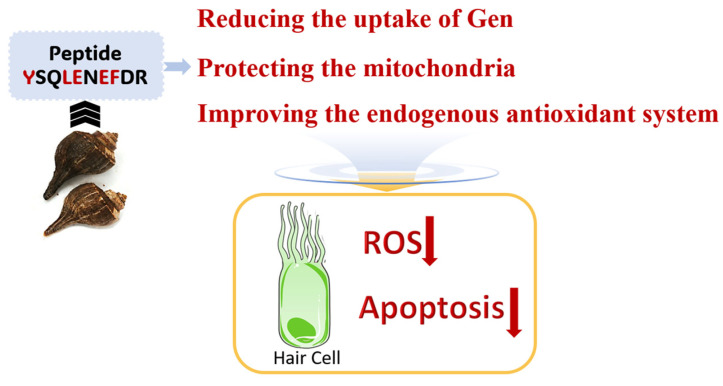
The proposed mechanism underlying the protective effect of the peptide from *Nac* on hair cells. The peptide reduces the uptake of Gen, protects the mitochondria, improves the endogenous antioxidant system to restrain the excessive accumulation of ROS, and then exerts protective effects against AmAn-induced hair cell damage. The structural characteristics of peptides may contribute to this biological process. *Nac*, *Neptunea arthritica cumingii*; Gen, gentamicin; ROS, reactive oxygen species; AmAn, aminoglycoside antibiotics.

Accumulating data indicate that hair cells are very susceptible to damage induced by AmAn. This susceptibility is related to the MET channel located at the top of hair cells [20]. Within the inner ear, only the hair cells exhibit properties of the MET channel. This channel is a nonselective cation channel. The narrowest diameter of the channel pore is estimated at 1.25 nm. The channel length is estimated at 3.1 nm, and the width of the pore mouth is <1.7 nm [21]. The MET channel is the main transporter for the entry of AmAn into hair cells. Meanwhile, the ototoxic effects of AmAn are initially dependent on the entry of drug molecules into the hair cells. Therefore, reducing the uptake of AmAn by targeting the MET channel may be more effective at stemming AmAn-induced hearing loss than other approaches. To our surprise, peptide YSQLENEFDR from *Nac* could reduce the accumulation of Gen in hair cells. Moreover, it did not impair the MET channels that rapidly open. We hypothesized that peptide YSQLENEFDR may be able to enter hair cells through MET channels such as Gen. There was competitive inhibition between Gen and the peptide. When hair cells were treated with both peptide YSQLENEFDR and Gen, the preferred entry of peptide into hair cells interfered with the entry of Gen. Finally, the activity of the MET channel was not affected, but the Gen in hair cells was reduced. That may be one of the underlying mechanisms that peptide YSQLENEFDR from *Nac* protects hair cells from AmAn (Figure 8).

The mitochondrial dysfunction and excessive accumulation of ROS in hair cells have been reported as the principal reasons that AmAn causes hair cell apoptosis [6]. In the current study, we found that peptide YSQLENEFDR from *Nac* could protect the mitochondria and restrain the excessive accumulation of ROS in hair cells. It also could rescue the abnormal expressions of *Mn-sod*, *Cu/Zn-sod*, *gstp1*, *gsto2*, and *cat*. The encoded proteins of the five aforementioned genes are all important members of the antioxidant defense system of cells [22]. For example, the encoded proteins of *Mn-sod* and *Cu/Zn-sod* can convert harmful superoxide radicals to molecular oxygen and hydrogen peroxide [23]. The encoded proteins of *gstp1* and *gsto2* are members of the glutathione *S*-transferase superfamily. They can scavenge the toxic accumulation of ROS [24]. The catalase encoded by *cat* can catalyze the decomposition of hydrogen peroxide into water and molecular oxygen [25]. Based on these facts, we assumed that the peptide from *Nac* may target the mitochondria and antioxidant-related genes at the same time. It prevented the production of ROS by maintaining mitochondrial homeostasis. Meanwhile, it also could improve the endogenous antioxidant system in hair cells by upregulating the expressions of *Mn-sod*, *Cu/Zn-sod*, *gstp1*, *gsto2*, and *cat* to quickly scavenge excess ROS. Corresponding to that, the incidence of apoptotic hair cell death induced by AmAn was reduced. That may be another mechanism of the otoprotective effects of peptide YSQLENEFDR (Figure 8).

It is well known that the physiological activity of the marine peptide is closely related to some structural characteristics of the peptide. For example, the antioxidant activity of peptides can be attributed to their amino acid compositions and sequences [26]. Hence, we supposed that the structural characteristic of peptide YSQLENEFDR may also contribute to protecting hair cells by scavenging ROS. The aromatic amino acids Tyr and Phe in the peptide may exert an antioxidant effect by donating protons to electron-deficient free radicals [27]. The occurrence of hydrophobic amino acid Leu may allow the peptide to interact with lipid-soluble free radicals and retard lipid peroxidation, which contributes to its antioxidant activity [28]. In addition, Glu is observed as an effective cation chelator that forms complexes with iron, zinc, and calcium, contributing to the antioxidant activity of the peptide [26]. Therefore, two glutamates in the peptide may also exert a significant protective effect on hair cells (Figure 8).

Collectively, our study suggested that the marine peptide YSQLENEFDR from *Nac* may be used in the development of drugs aimed at preventing and treating the hearing loss induced by AmAn. Definitely, several limitations to the present study should also be considered. First, the otoprotective effect of the peptide should be verified in mammalian models such as mouse or rat. Second, while the mitochondrial protective activity of peptide had been observed, the underlying mechanisms need to be further studied, which we are currently planning.

## 4. Materials and Methods

### 4.1. Chemicals and Reagents

This study was performed at the Key Laboratory for Drug Screening Technology of the Shandong Academy of Sciences in 2023–2024.

Gen was purchased from MDBio (Qingdao, China). YO-Pro-1, CellROX Deep Red Reagent, and FM1-43 were acquired from Thermo Fisher Scientific (Waltham, MA, USA). Electron microscope fixation liquid was obtained from Servicebio (Wuhan, China). Succinimidyl esters of Texas Red were procured from AAT Bioquest (Sunnyvale, CA, USA). TUNEL BrightRed Apoptosis Detection Kit, FastPure Cell/Tissue Total RNA Isolation Mini Kit, HiScript III 1st Strand cDNA Synthesis Kit, and AceQ Universal SYBR qPCR Master Mix were acquired from Vazyme Biotech Co., Ltd. (Nanjing, China). The Mitochondrial Permeability Transition Pore Assay Kit was obtained from Beyotime (Shanghai, China).

### 4.2. Preparation of the Peptide

Peptide YSQLENEFDR was first isolated from *Nac* by an activity-directed separation technique in our laboratory [9]. In this study, the synthetic peptide of YSQLENEFDR was used with a purity of 95% (CelLmano Biotech Co., Ltd., Hefei, China).

### 4.3. Animals

Adult wild-type AB strain zebrafish larvae were kept under a constant temperature (28 °C ± 0.5 °C) and a 14-h light/10-h dark photoperiod at the Key Laboratory for Drug Screening Technology of the Shandong Academy of Sciences (Jinan, China). The zebrafish larvae were obtained from natural mating. Larvae at 5 dpf were used for animal experiments.

### 4.4. Sample Treatments

Zebrafish larvae at 5 dpf were randomly transferred to six-well cell culture plates with a density of approximately 15 larvae per well. Then, they were subjected to the following three treatments. Larvae in the control group were incubated in embryo medium [0.5 mM KCl, 15 mM NaCl, 1 mM CaCl_2_, 1 mM MgSO_4_, 0.05 mM (NH_4_)_3_PO_4_, 0.15 mM KH_2_PO_4_, and 0.7 mM NaHCO_3_] for 2 h. Larvae in the Gen exposure group were preincubated in embryo medium for 1 h. Subsequently, they were incubated with 125 μM Gen for 1 h. Larvae in the peptide group were preincubated with different concentrations of peptide YSQLENEFDR (10, 15, and 20 μg/mL) for 1 h. Then, they were co-incubated with 125 μM Gen and different concentrations of the peptide (10, 15, and 20 μg/mL) for 1 h (Figure 1B).

### 4.5. Hair Cell Assessment

The larvae from each group were cleaned using the embryo medium. They were then treated with 2 μM YO-Pro-1 for 30 min in the dark and anesthetized. Hair cells were easily stained by the fluorescent dye YO-Pro-1. Lateral line neuromast hair cells on one side of each larva were photographed using a fluorescence microscope (AXIO Zoom V16, Zeiss, Dresden, Germany). The hair cell number was quantified in seven neuromasts (O1, O2, MI1, LII1, LII3, L4, and L5) per larva [29,30].

### 4.6. Scanning Electron Microscopy

After treatment with both Gen and peptide YSQLENEFDR, zebrafish larvae were fixed in electron microscope fixation liquid and dehydrated using a series of ethanol and isoamyl acetate solutions, each for 15 min: 30% ethanol, 50% ethanol, 70% ethanol, 80% ethanol, 90% ethanol, 100% ethanol, and 100% isoamyl acetate. Subsequently, they were dried in a freeze dryer (K850, Quorum, Laughton, East Sussex, UK) and coated with platinum using a sputter coater (MC1000, HITACHI, Tokyo, Japan). Images were taken using a scanning electron microscope (SU8100, HITACHI, Tokyo, Japan).

### 4.7. Detection of Gen Uptake

Succinimidyl esters of Texas Red at a concentration of 2 mg/mL and Gen were agitated together overnight to produce the GTTR which was the Gen tagged with red fluorescence [14]. Larvae were subjected to Gen exposure and treatment with different concentrations of the peptide (10, 15, and 20 μg/mL). They were treated as presented in Figure 1B, but Gen was replaced by GTTR. Then, larvae were rinsed in embryo medium and anesthetized. A confocal scanning laser microscope (FV1000, Olympus, Tokyo, Japan) was used to observe the larvae. The fluorescence intensities were measured using Image-Pro Plus 5.1.

### 4.8. FM 1-43 Uptake Assay

After being treated with different concentrations of peptide YSQLENEFDR (0, 10, 15, and 20 μg/mL) for 2 h, larvae were incubated with 5 μM FM1-43 which was a fluorescent dye and could specifically enter hair cells through MET channels for 1 min and rinsed in fresh embryo medium three times. Then, they were anesthetized and photographed using a fluorescence microscope (SZX16, Olympus, Tokyo, Japan). The fluorescence intensities were measured using Image-Pro Plus 5.1.

### 4.9. Determination of ROS Levels

Zebrafish larvae were treated as presented in Figure 1B. Next, ROS levels in hair cells were detected using the CellROX Deep Red Reagent Kit following the manufacturer’s instructions with a slight modification. In brief, larvae were incubated with 5 μM CellROX Regent which was the fluorogenic probe measuring ROS in live cells for 100 min at 28.5 °C in the dark. Then, they were anesthetized and photographed using a fluorescence microscope (IX51, Olympus, Tokyo, Japan).

### 4.10. Apoptosis Assessment

Apoptotic hair cells were assessed using the terminal deoxynucleotidyl transferase (TdT)-mediated dUTP-biotin nick end-labeling (TUNEL) assay with a TUNEL BrightRed Apoptosis Detection Kit. In short, the paraformaldehyde-fixed zebrafish larvae were incubated in a TUNEL reaction mixture at 37 °C for 90 min. Subsequently, they were photographed using a confocal scanning laser microscope (FV1000, Olympus, Tokyo, Japan).

### 4.11. mPTP Measurements

The opening of mPTP was detected using the Mitochondrial Permeability Transition Pore Assay Kit according to the manufacturer’s instructions with a slight modification, as follows. Zebrafish larvae were incubated with fluorescence quenching solution for 90 min, and then the incubated liquid was changed to fresh embryo medium for 90 min under low light conditions. After being washed with fresh embryo medium, the fluorescence was observed using a fluorescence microscope (AXIO Zoom V16, Zeiss, Dresden, Germany). When mPTP was closed, calcein was able to load onto the mitochondria, resulting in the presentation of green fluorescence. When mPTP was open, calcein could not load onto the mitochondria, resulting in the disappearance of green fluorescence.

### 4.12. Detection of Gene Expression

The expressions of five genes (*Mn-sod*, *Cu/Zn-sod*, *gstp1*, *gsto2*, and *cat*) were detected using RT-qPCR. The total RNA was extracted from the larval tissue using the FastPure Cell/Tissue Total RNA Isolation Mini Kit according to the manufacturer’s instructions. Next, RNA was reverse transcribed into cDNA using the HiScript III 1st Strand cDNA Synthesis Kit. The RT-qPCR was conducted using the AceQ Universal SYBR qPCR Master Mix. The housekeeping gene *β-actin* was used as a reference gene. The primer sequences of the aforementioned genes are presented in Table 1.

### 4.13. Statistical Analysis

Data were presented as mean ± standard error of the mean and analyzed using ANOVA. *p* < 0.05 was considered statistically significant.

## 5. Conclusions

Our study revealed that peptide YSQLENEFDR from *Nac* could protect hair cells from Gen-induced hair cell damage in zebrafish. The underlying mechanism may be that the peptide blocked the cellular entry of Gen by competitive inhibition and prevented ROS accumulation by protecting the mitochondria and improving the endogenous antioxidant system in hair cells. The amino acid compositions of the peptide may contribute to this biological process. However, these mechanisms should be further verified in other animal models. Taken together, our data implied that the marine peptides have the potential to prevent AmAn-induced hearing loss.

## Figures and Tables

**Table 1:** The sequences of primer pairs used in RT-qPCR assay.

No	Gene Symbol	Forward Primer	Reverse Primer
1	*Mn-sod*	TAGATGTCTGGGAACATGCG	TGGCTTTAACATAGTCCGGTC
2	*Cu/Zn-sod*	GGTGGCAATGAGGAAAGTC	ATCACTCCACAGGCCAGA
3	*gstp1*	CTACAACCTGTTCGATCTCCT	GGGCAGAGATCTTGTCCAC
4	*gsto2*	ATGGCTTCATCTCCAAAATGC	AGGGCAGAATCTCATGCTGTAG
5	*cat*	AGGGCAACTGGGATCTTACA	GATCCTTCAGGTGAGTCTGC
6	*β-actin*	AGAGTATGAGCTGCCTGACG	CCGCAAGATTCCATACCCA

## Data Availability

The data presented in the current study are available on request from the corresponding author.
